# *Linderae radix* ethanol extract attenuates alcoholic liver injury via attenuating inflammation and regulating gut microbiota in rats

**DOI:** 10.1590/1414-431X20197628

**Published:** 2019-05-16

**Authors:** Zhaohuan Lou, Junwei Wang, Yingjun Chen, Chandi Xu, Xinyi Chen, Tiejuan Shao, Kena Zhang, Hongying Pan

**Affiliations:** 1 Institute of Medical Material, Zhejiang Chinese Medical University, Hangzhou, Zhejiang, China; 2 Emergency Department, People's Hospital of Tiantai County, Tiantai, Zhejiang, China; 3 Department of Infectious Diseases, People's Hospital of Tiantai County, Tiantai, Zhejiang, China; 4 School of Clinical Medicine Sciences, Zhejiang Chinese Medical University, Hangzhou, Zhejiang, China; 5 School of Basic Medical Sciences, Zhejiang Chinese Medical University, Hangzhou, Zhejiang, China; 6 Department of Infectious Diseases, Zhejiang Provincial People's Hospital, Hangzhou, Zhejiang, China

**Keywords:** *Lindera aggregata* (Sims) Kosterm, Ethanol extract, Alcoholic liver disease, Intestinal endotoxemia, 16S ribosomal RNA gene sequencing, Tight junction protein

## Abstract

This study aimed to explore the influence of gut microbiota alterations induced by *Linderae radix* ethanol extract (LREE) on alcoholic liver disease (ALD) in rats and to study the anti-inflammatory effect of LREE on ALD through the lipopolysaccharide (LPS) toll-like receptor 4 (TLR4)-nuclear factor kappa B (NF-κB) pathway. ALD rat models were established by intragastric liquor [50% (v/v) ethanol] administration at 10 mL/kg body weight for 20 days. Rats were divided into six groups: normal group (no treatment), model group (ALD rats), Essentiale group (ALD rats fed with Essentiale, 137 mg/kg), and LREE high/moderate/low dose groups (ALD rats fed with 4, 2, or 1 g LREE/kg). NF-κB and LPS levels were evaluated. Liver pathological changes and intestinal ultrastructure were examined by hematoxylin and eosin staining and transmission electron microscopy. The gut microbiota composition was evaluated by 16S rDNA sequencing. Expression levels of TLR4 and CD68 in liver tissue, and occludin and claudin-1 in intestinal tissue were measured. LREE treatment significantly reduced NF-κB and LPS levels, improved liver pathological changes, and ameliorated intestinal ultrastructure injury. Meanwhile, LREE-fed groups showed a higher abundance of *Firmicutes* and a lower abundance of *Bacteroidetes* than the rats in the model group. Administration of LREE suppressed TLR4 overexpression and promoted the expression of occludin and claudin-1 in intestine tissue. Thus, LREE could partly ameliorate microflora dysbiosis, suppress the inflammatory response, and attenuate liver injury in ALD rats. The protective effect of LREE might be related to the LPS-TLR4-NF-κB pathway.

## Introduction

Currently, alcohol has become a major pathogenic factor for liver disease (1). According to a report provided by the World Health Organization in 2014, every year approximately 3.3 million deaths are attributed to harmful alcohol consumption worldwide (2). The pathogenesis of alcohol-induced liver injury involves the formation of reactive aldehydes free radicals resulting from ethanol metabolism (3). Alcoholic liver disease (ALD) is a common cause of chronic liver injury, resulting from increased alcohol intake (4). Patients with ALD frequently progress to liver fibrosis and cirrhosis, which have increased risks of complications, such as portal hypertension, and liver cancer (5). The pathogenesis of ALD is complicated and unclear, which has led to the main cornerstone of ALD treatment being based only on lifestyle modification, including abstinence, nutritional therapy, and corticosteroid therapy. Therefore, it is crucial to investigate the pathogenesis of ALD and to develop new therapeutic strategies for ALD.

Intestinal endotoxemia (6) and the imbalance of the gut-liver axis are involved in ALD. The imbalance of the gut-liver axis, which induces an inflammatory response, plays a vital role in the progression of ALD (7). The small intestine, as the main organ responsible for absorption (8), contains approximately 10^14^ microorganisms (9). The Bacteroidetes and the Firmicutes are in balance under physiological conditions (10,11). Excessive alcohol intake damages the balance of gut microbiota (12), which will affect intestinal permeability (13) and increase the circulating lipopolysaccharide (LPS) concentration (14). This gut-derived LPS can promote liver injury and aggravate the inflammatory response (10,14,15). Thus, regulating gut microbiota to inhibit the trigger of inflammation might be the effective way to ease the progression of ALD.

LPS, also named endotoxin, is recognized by toll-like receptor 4 (TLR4), together with myeloid differentiation protein 2, lipopolysaccharide receptor CD14, and LPS-binding protein (8,15). The LPS-MD2/TLR4 complex then leads to the activation of the myeloid differentiation factor 88-dependent pathway (7,15). This pathway can induce nuclear factor-kappa B (NF-κB) activation, which has an important effect on the inflammatory response in the liver. The expression of inflammatory cytokines, especially tumor necrosis factor-α (TNF-α), is regulated by NF-κB (7,16). Therefore, as a bridge, LPS-TLR4-NF-κB, which links the gut microbiota and inflammation, may provide a novel target for ALD treatment.

*Linderae radix* is a traditional Chinese medicine extracted from the tubers of *Lindera aggregata* (Sims) Kosterm, which contains sesquiterpene lactones (17) and alkaloids (18), and has anti-inflammatory effects by inhibiting NF-κB and mitogen-activated protein kinase activation (19). *Linderae radix* also contains furan sesquiterpene components, which could lower the serum activities of transaminases and suppress hepatocyte fatty infiltration in liver injury models (20). *Linderae radix* water extracts showed dose-dependent cytoprotective activity against ethanol-induced acute gastric injury by mediating endogenous prostaglandins release (21). In a previous study, we evaluated the therapeutic effects of *Linderae radix* extracts, such as water and ethanol extracts, on alcoholic liver injury, and found that the protective effect of the ethanol extract was superior to that of the water extract (22). This study aimed to determine the protective effect of an *Linderae radix* ethanol extract (LREE) on ALD model rats and whether this protective effect was related to the LPS-TLR4-NF-κB pathway.

## Material and Methods

### Materials

**Figure 1.**
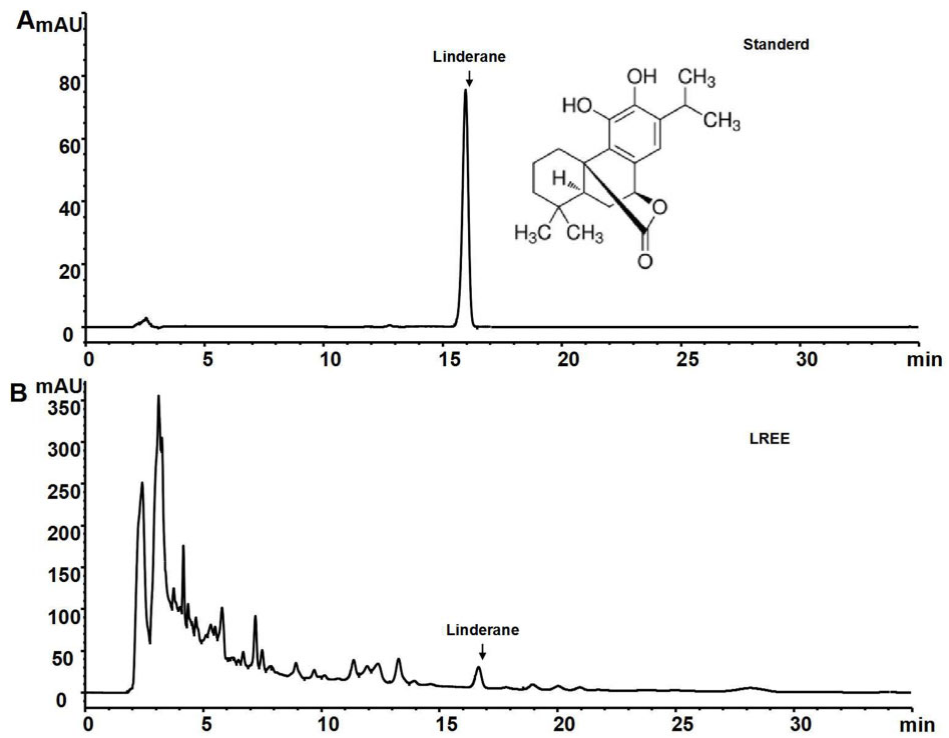
High performance liquid chromatography chromatogram (HPLC). **A**, The HPLC profile of standard Linderane is shown with a peak around 16 min. **B**, The HPLC profile of *Linderae radix* ethanol extract (LREE) is presented with a peak at 16.3 min. Linderane and LREE had similar retention times, which showed they might be homologs.

Prepared slices of *Linderae radix*, which were supplied by Zhejiang Tiantaishan Wuyao Biological Engineering Co., Ltd. (China), were identified by Professor Kong-rong Chen, who is an expert on plants and works in the College of Pharmacy of Zhejiang Chinese Medical University (China). LREE was extracted using 75% (v/v) ethanol, and the quality of the LREE was detected using high performance liquid chromatography with the standard Linderane (Figure 1).

### Animals and treatments

The animal experiments were approved by the Animal Ethics Committee of Zhejiang Academy of Medical Sciences (No. ZSLL-2015-124). Thirty male Sprague-Dawley rats (160–180 g) were obtained from the Experimental Animal Center of Zhejiang Academy of Medical Sciences (China). Rats were kept in an environmentally controlled breeding room (temperature: 25±1°C, humidity: 55±5%, light/dark cycle: 12/12 h) for 7 days before the experiment.

ALD model rats were established by the intragastric administration of liquor containing 50% (vol/vol) alcohol, 48–49% water, and 1–2% aroma compounds (Beijing Red Star Co., Ltd., China) at 10 mL/kg body weight, once daily for 20 days. Rats were randomly separated into six groups: normal group (Normal, n=5, rats were intragastrically administered distilled water at 10 mL/kg body weight per day for 20 days, without any other treatment), model group (Model, n=5, ALD model rats without any treatment), positive control group (Essentiale group, n=5, ALD model rats were fed with Essentiale; 137 mg/kg, Sanofi-Aventis Beijing Pharmaceutical Co., Ltd., China), LREE high dose group (LGH group, n=5, ALD model rats fed with 4 g/kg LREE), LREE moderate dose group (LGM, n=5, ALD model rats fed with 2 g/kg LREE), and LREE low dose group (LGL, n=5, ALD model rats fed with 1 g/kg LREE). The dosages of LREE extract provided to the animals in the present study was referenced from the equivalent dose conversion of 10 g/60 kg for humans provided in the Chinese Pharmacopoeia (2015 version, http://www.drugfuture.com/standard/), as well as referenced from our previous study on the dosage and function association of LREE against ALD in mice, in which the maximum effect of LREE against ALD was obtained at a dose between 2–4 g/kg (23). Food and water were administered *ad libitum* during the period of the experiment for the rats in all groups. Finally, all rats were anesthetized by intraperitoneal injection of 10% chloral hydrate (2 mL/kg) and decapitated. Blood was collected from the abdominal aorta before death. Serum was separated to detect enzymes and inflammatory cytokines. The left lobe of the liver was sampled for histopathology and western blotting analysis.

### Hepatic histopathological observation

The left lobe of the liver was used to observe the hepatic histopathology. Sections (5-mm thick) were 10% neutral formalin-fixed, 70–100% ethyl alcohol gradient-dehydrated, and paraffin-embedded before being stained with hematoxylin and eosin. Pathological changes were investigated using a biomicroscope (B5-223IEP, Motic China Group Co., Ltd., China) coupled with an Advanced 3.2 Image Analysis System (Motic China Group Co., LTD., China).

### Biochemical assays and enzyme-linked immunosorbent assay

The serum concentrations of aspartate aminotransferase (AST) and alanine aminotransferase (ALT) were measured using a fully automatic blood biochemistry analyzer (TBA-120FR, Toshiba Medical System Co. Ltd., Japan) and the total bilirubin (T.BIL) level was measured using a caffeine colorimetric method (Ningbo Meikang Biota Technology Co., Ltd., China). Serum levels of interleukin (IL)-8, IL-6, NF-κB, TNF-α, and LPS were analyzed using enzyme-linked immunosorbent assay (ELISA) kits (Shanghai Yuanye Bio Technology Co., Ltd., China), according to the manufacturer's instructions.

### Transmission electron microscopy analysis of intestinal tissue

Glutaraldehyde (2.5%) was used to fix the specimens, which were then rinsed in 0.1 mol/L phosphate buffer. A graded ethanol series was then used to dehydrate the tissue. Ultra-thin slices were then cut and examined in a transmission electron microscope (Hitachi-7650, Japan).

### Fecal DNA extraction and 16S rDNA amplification

DNA was extracted from fecal samples using a Fast DNA SPIN kit for feces (Beijing Think-Far Technology Co., Ltd, China). The V3–V4 region of the bacterial 16S rDNA was amplified using miseq adapter-linked and bar-coded universal primers [forward primer: 5′-CAAGCAGAAGACGGCATACGAGATGTGACTGGAGTTCAGACGTGTGCTCTTCCGATCT(barcode)ACTCCTACGGGAGGCAGCAG-3′; reverse primer: 5′-AATGATACGGCGACCACCGAGATCTACACTCTTTCCCTACACGACGCTCTTCCGATCTX(barcode)GGACTACHVGGGTWTCTAAT-3′]. A 50-μL reaction system, including 25 μL of PCR Master Mix Buffer (Finnzymes, Finland), 3 μL of dimethyl sulfonic acid, 3 μL of each primer, 10 μL of template DNA, and nuclease free water, was subjected to the following cycling conditions: 98°C for 30 s, followed by 30 cycles of 98°C for 15 s, 58°C for 15 s, and 72°C for 15 s; plus 72°C for 1 min as the final elongation step. PCR products were purified using an AXYGEN Gel Extraction Kit (AXYGEN, USA).

### 16S rDNA sequencing and analysis

The purified PCR products were sequenced on the Illumina^®^ Miseq platform (USA). The raw sequencing reads were filtered using QIIME 1.8.0 (24) according to the following criteria: 1) no ambiguous bases; 2) exact matches to the bar code tags; and 3) removal of low-quality scores in a sliding window and reserving the sequences of lengths more than 50 bp. Then, the valid sequences were aligned to the 16S Microbial.tar.gz database (ftp://ftp.ncbi.nih.gov/blast/db/). The taxonomic classification of the sequence reads was identified by the ribosomal database project (RDP) classifier (25). Operational taxonomic units (OTUs) at 97% similarity were determined using UCLUST (http://drive5.com/usearch/manual/uclust_algo.html) (26). The estimated coverage of the libraries was calculated using the formula: C=(1−n_1_/N)×100%, where N is the total number of clones that were analyzed in the library, n_1_ is the number of OTUs containing only one sequence, and C is the homologous coverage (27). Rarefaction curves were generated using RarefactWin (University of Georgia, http://www.uga.edu/-strata/AnRareReadme.html). The diversity indices (Shannon-Wiener, ACE, and Chao1 index) for the samples were calculated using QIIME (version 1.8.0) (24). An image representing the relative abundance of each microbe was drawn using Origin software (version: 8.0; OriginLab, USA).

### Western blotting analysis for CD68 and TLR4

Proteins were extracted from the liver and quantified using the bicinchoninic acid method. First, 10% sodium dodecyl sulfate polyacrylamide gel electrophoresis was applied to separate the proteins, and then the proteins were transferred onto polyvinylidene fluoride membranes (Millipore, USA). The membranes were incubated with anti-TLR4 and anti-CD68 (dilution for both: 1:100; SC-12511, Santa Cruz Biotechnology, USA) overnight at 4°C. The membranes were then incubated with horseradish peroxidase-conjugated Donkey anti-goat IgG-B and Donkey anti-mouse IgG-B (Santa Cruz Biotechnology), respectively, for 1 h at 37°C. The membranes were reacted with enhanced chemiluminescence substrate solution (Millipore) and detected using a chemiluminescence gel imaging system (Peiqing Science & Technology, China).

### Determination of occludin and claudin-1 protein levels in intestinal tissue

Intestinal tissues were embedded in paraffin wax and cut into slices. The slices were deparaffinized, hydrated, and the antigens were repaired, and endogenous peroxidase was inactivated. The sections were incubated with primary antibodies (anti-occludin, dilution 1:100, Novus, USA; anti-claudin-1, dilution 1:100, Abcam, Cambridge, UK) overnight at 4°C, washed with phosphate-buffered saline and incubated with the secondary antibody (horseradish peroxidase-rabbit anti-goat IgG, Santa Cruz Biotechnology) and Streptavidin-Biotin Complex, successively. Afterwards, 3,3′-diaminobenzidine and hydrogen peroxide were applied to visualize the labeling. Finally, the slices were observed using Image-Pro plus 5.1 (US IPP Image Analysis Software Co., USA) to analyze the image, select the specific area in the image, and calculate the integrated optical density. The integrated optical density value was obtained from the mean value of five fields in the same slice and three slices were measured for each specimen.

### Statistical analysis

All data are reported as means±SD. Means between different groups were compared using analysis of variance (ANOVA), followed by Tukey's *post hoc* test for multiple comparisons, calculated using the SPSS 16.0 software (IBM, USA). P<0.05 was considered statistically significant.

## Results

### LREE ameliorated the liver injury induced by alcohol

**Figure 2.**
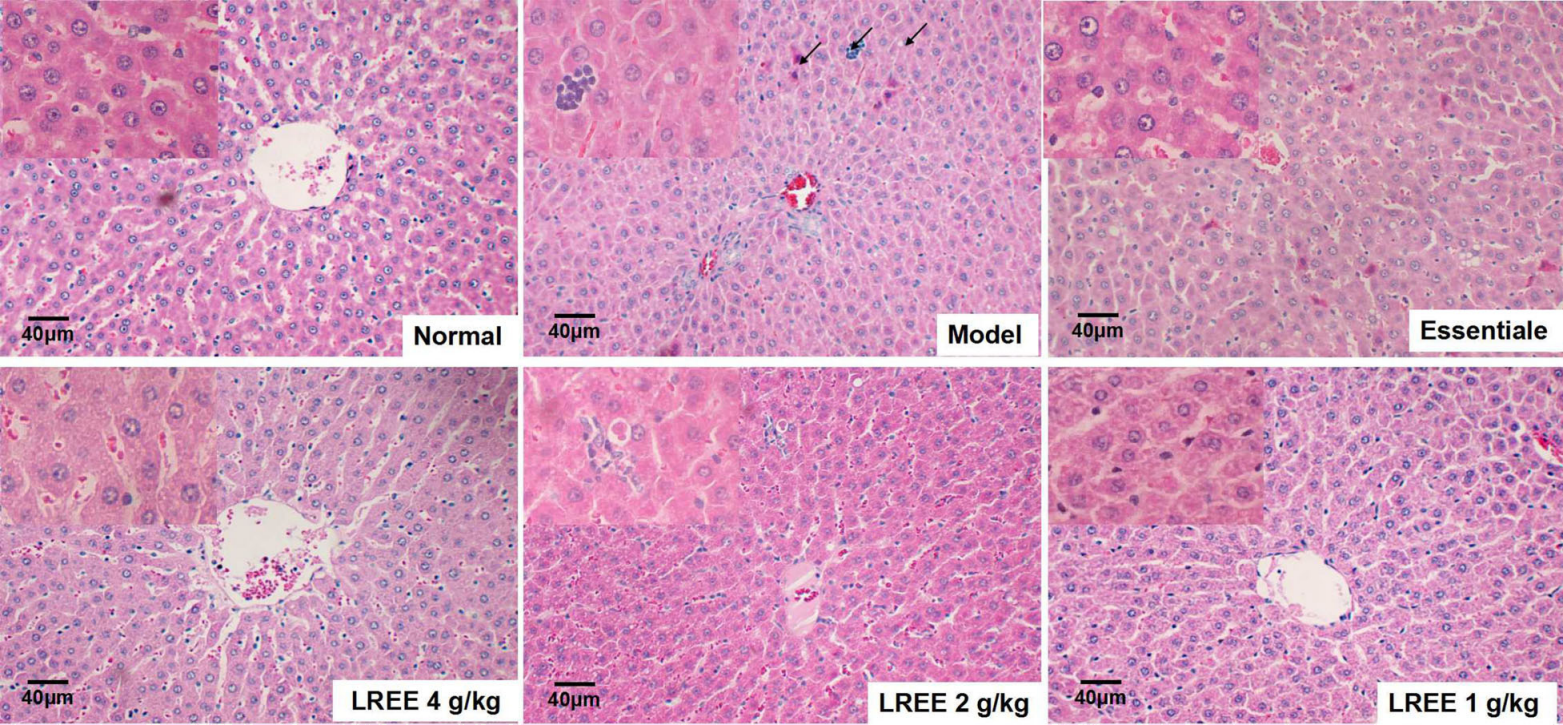
Optical microscope findings in the liver (H&E, ×200, bar: 40 μm). To induce alcoholic liver disease, 50% alcohol was provided, and *Linderae radix* ethanol extract (LREE) was administered daily by gavage for the same period. Inflammatory cells clusters and associated necrosis were observed in the livers of model rats. The arrows indicate neutrophil infiltration.

Serum ALT and T.BIL levels increased significantly in the model group (P<0.05), but remained lower in the LREE groups (P<0.05). Although there were no significant changes in AST level in serum among the normal and model groups (P>0.05), the AST levels were decreased significantly in LGL group compared with that in model group (P<0.05, Table 1). Normal cells showed lobular architectures, but increased inflammatory cell infiltration was observed in the model group. In contrast, LREE ameliorated these pathological changes (Figure 2).

**Table 1.** Serum levels of alanine aminotransferase (ALT), aspartate aminotransferase (AST), and total bilirubin (T.BIL) in the different groups.

Group	ALT (U/L)	AST (U/L)	T.BIL (μmol/L)
Normal	50.3±9.0	137.9±27.3	7.62±1.20
Model	74.2±13.4^++^	153.4±29.2	9.38±2.04^+^
Essentiale	66.2±12.4	153.0±36.3	6.60±1.61**
LREE (4 g/kg)	60.0±16.4*	145.0±47.6	7.14±2.11*
LREE (2 g/kg)	57.9±10.5**	167.1±61.7	6.83±1.41**
LREE (1 g/kg)	59.4±9.7**	131.9±21.5*	6.69±1.28**

Data are reported as means±SD. Except the normal group, other groups were administered 10 mL/kg body weight 50% (v/v) of liquor intragastrically once daily for 20 days. Normal group (NC): administered an equal volume of distilled water; Model control group (MC): rats induced to develop alcoholic liver disease; Essentiale group: model rats fed with 137 mg/kg Essentiale (positive control); LREE: model rats treated with different concentrations of *Linderae radix* ethanol extract (LREE). ^+^P<0.05 and ^++^P<0.01 *vs* NC; *P<0.05 and **P*<*0.01 *vs* MC (ANOVA).

### LREE decreased the levels of inflammatory cytokines

The levels of inflammatory cytokines in each group are shown in Table 2. Compared with those in the normal group, serum levels of IL-6 (P<0.01), TNF-α (P<0.01), NF-κB (P<0.01), endotoxin (P<0.01), and IL-8 (P<0.05) in the model group increased significantly. Compared with those in the model group, the levels of IL-8 and NF-κB were significantly decreased in the LGH group (P<0.01), while serum TNF-α and NF-κB levels were significantly decreased in the LGM group, (P<0.01). Meanwhile, the endotoxin level in the model group was significantly higher than that in the normal group (P<0.01). Each dose of LREE could significantly downregulate the endotoxin level (P<0.01) compared with that in the model group. Notably, the serum levels of IL-6 and IL-8 were gradually reduced in the LGL, LGM, and LGH groups; however, the TNF-α, NF-κB, and endotoxin levels did not show a dose-response relationship in the different LREE concentrations. In addition, the anti-inflammatory effect of LREE was best in the LGM group (2 g/kg), which showed significantly reduced levels of ALT, T.BIL, IL-8, NF-κB, and TNF-α.

**Table 2.** Serum inflammatory cytokines levels of interleukin (IL)-6, IL-8, tumor necrosis factor (TNF)-α, nuclear factor kappa B (NF-kB), and hepatic portal vein endotoxin level.

Group	Serum IL-6 (pg/mL)	Serum IL-8 (pg/mL)	Serum TNF-α (pg/mL)	Serum NF-κB (pg/mL)	Endotoxin in portal vein (EU/mL)
Normal	160.9±18.5	200.7±14.6	276.8±21.9	903.2±37.5	55.3±4.8
Model	190.0±9.4^++^	213.3±13.9^+^	334.2±11.5^++^	975.0±50.9^++^	68.3±3.3^++^
Essentiale	174.0±26.0*	177.8±26.4**	320.0±31.0	906.8±81.1*	62.1±8.1*
LREE (4 g/kg)	162.4±24.6**	187.1±12.6**	320.4±23.4	907.6±54.6**	56.1±4.5**
LREE (2 g/kg)	187.6±19.4^#^	200.6±16.3*^#^	304.6±28.7**	890.7±52.8**	59.9±4.3**^#^
LREE (1 g/kg)	185.6±33.1^#^	209.5±20.7^##^	292.5±22.5**^##^	949.6±84.2	54.9±4.5**

Data are reported as means±SD. ^+^P<0.05 and ^++^P*<*0.01 *vs* normal control; *P<0.05 and **P<0.01 *vs* model control; and ^#^P<0.05 and ^##^P<0.01 *vs* 4 g/kg *Linderae radix* ethanol extract (LREE) group (ANOVA).

### LREE ameliorated the intestinal ultrastructure injury induced by alcohol

**Figure 3.**
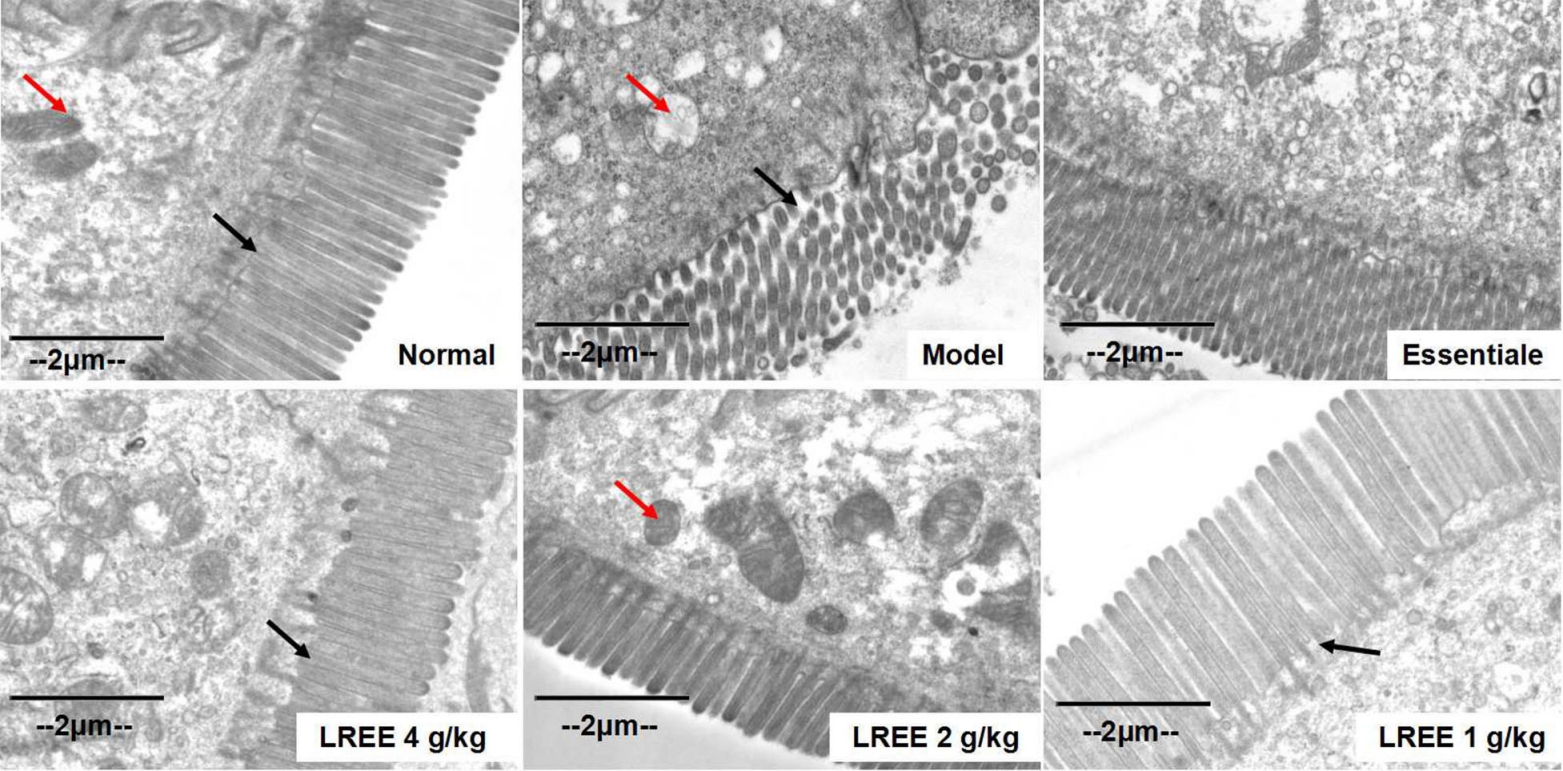
Results of transmission electron microscopy of small intestine tissue (×12000, bar 2 μm). To induce alcoholic liver disease, 50% alcohol was provided, and *Linderae radix* ethanol extract (LREE) was administered daily by gavage for the same time. Alcohol caused irregular and deteriorated microvilli (black arrows), and swelling and vacuolar degeneration in mitochondria (red arrows). Tufted microvilli and improved mitochondria were observed in the LREE groups.

The transmission electron microscopy results are shown in Figure 3. In the normal group, microvilli in the small intestinal epithelium were arranged regularly, and tight junctions and mitochondria were undamaged. In the model group, irregular and sparse microvilli were observed and mitochondria with vacuolar degeneration were found. Compared with the damage observed in the model control rats, more tufted microvilli were found in the LREE-treated rats, and less vacuolar degeneration in the mitochondria was observed.

### Characterization of gut microbiota in fecal samples

The values of library coverage (C) ranged from 92 to 93.2% in the different groups (Table 3), revealing that more than 92% of the 16S rDNAs were captured in all libraries.

The Shannon diversity index in the normal group was lower than that in the model group; however, the Shannon diversity index in the Essentiale, LGH, LGM, and LGL groups was higher than that in the model group. Meanwhile, the Shannon diversity index in LGL group was higher than that in the other groups (Table 3).

**Table 3.** Diversity indexes for bacteria in rat feces in different groups.

Group name	Chao1	Shannon	Ace	Simpson	Coverage (%)
Normal	5008.58±1521.17	7.05±0.83	5421.66±1641.78	0.93±0.04	0.92±0.02
Model	6729.16±1907.13	7.37±1.46	7347.58±2121.65	0.93±0.08	0.93±0.02
Essentiale	5789.35±1246.32	8.12±0.44	6250.88±1356.77	0.98±0.02	0.93±0.03
LREE (4 g/kg)	6724.48±1416.95	8.31±0.32	7304.70±1607.29	0.98±0.01	0.92±0.01
LREE (2 g/kg)	6634.53±1793.71	8.17±0.94	7280.06±1984.69	0.97±0.04	0.93±0.02
LREE (1 g/kg)	6798.61±904.75	8.49±0.50	7370.93±1160.95	0.99±0.01	0.93±0.02

Data are reported as means±SD. Except the normal group, other groups were administered 10 mL/kg body weight 50% (v/v) of liquor intragastrically once daily for 20 days. Normal group: administered an equal volume of distilled water; Model group: rats induced to develop alcoholic liver disease; Essentiale group: model rats fed with 137 mg/kg Essentiale (positive control); LREE: model rats treated with *Linderae radix* ethanol extract (LREE). There were no significant differences among these data (ANOVA).

Microbiota in the feces at the level of phylum, class, order, family, and genus are presented in Table 4. Compared with the Essentiale group, Bacteroidetes (at the phylum and class levels), Bacteroidales (at the order level), Prevotellaceae (at the family level), and Prevotella (at the genus level) were increased, while Firmicutes, Spirochaete (at the phylum level), Actinobacteria, Spirochaetes (at the class level), Clostridiales, Lactobacillales (at the order level), Ruminococcaceae, Lachnospiraceae, Lactobacillales (at the family level), Lactobacillus, and Oscillospira, Treponema and Ruminococcus (at the genus level) were decreased in the model group. Furthermore, compared with that in the model group, the relative abundance of Firmicutes (at the phylum level) and Lactobacillales (at the order level) was increased in the LGL and LGM groups. Compared with that in the LGL and LGM groups, the relative abundance of Actinobacteria and Spirochaetes at the class level was increased in the LGH group. Interestingly, LREE treatment increased the abundance of Clostridiales (at the order level), Ruminococcaceae, and Lactobacillales (at the family level), and decreased the abundance of Bacteroidetes (at the order level) and Prevotellaceae (at the family level) vs the model group. Moreover, LREE treatment increased the abundance of Lactobacillus, Oscillospira, Treponema, and Ruminococcus at the genus level. Among them, the abundance of Lactobacillus and Ruminococcus increased substantially in the LGM group, while Treponema and Oscillospira were increased in the LGH and LGL groups, respectively.

**Table 4.** Effects of LREE on relative bacterial abundance of gut microbiota in feces of rats.

Group	Phylum	Class	Order	Family	Genus
Normal	2018.2±714.9	2018.2±714.9	2017.8±714.8	1695.6±611.5	1069.8±368.6
Model	2501.4±778.5	2501.2±778.4	2499.6±778.4	2121.4±665.8	1178.4±394.2
Essentiale	2268.2±547.9	2268.2±547.9	2267.8±547.6	1712.0±392.0	811.2±217.6
LREE (4 g/kg)	2621.6±593.8	2621.4±593.5	2619.8±593.9	2053.0±527.9	1123.2±416.8
LREE (2 g/kg)	2632.2±613.9	2631.4±614.6	2630.6±615.1	2114.8±499.2	1138.0±330.7
LREE (1 g/kg)	2749.0±347.6	2748.8±347.3	2747.4±347.7	2153.8±240.3	1100.0±203.6

Data are reported as means±SD. Except the normal group, other groups were administered 10 mL/kg body weight 50% (v/v) of liquor intragastrically once daily for 20 days. Normal group: administered an equal volume of distilled water; Model group: rats induced to develop alcoholic liver disease; Essentiale group: model rats fed with 137 mg/kg Essentiale (positive control); LREE: model rats treated with *Linderae radix* ethanol extract (LREE). There were no significant differences among these data (ANOVA).

### LREE decreased the expression of TLR4 and CD68

**Figure 4.**
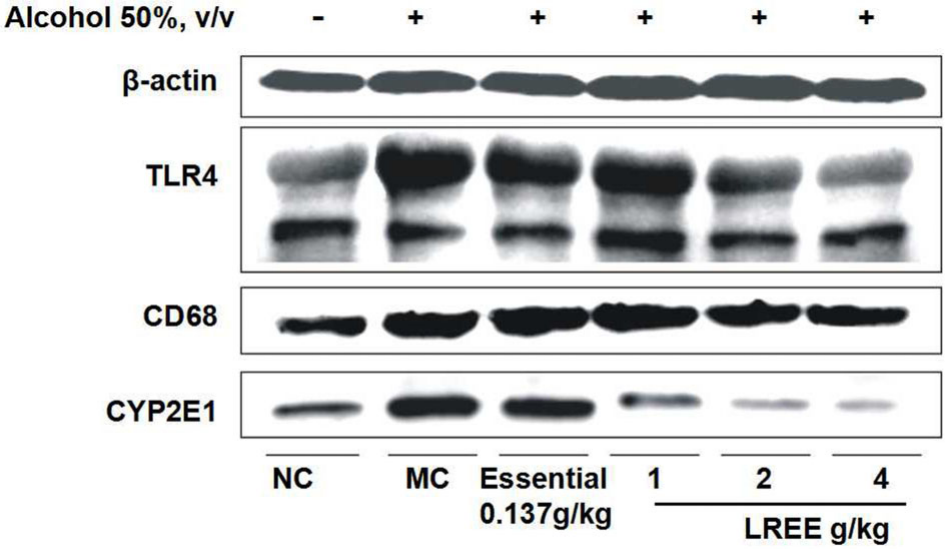
Western blotting analysis of cluster of differentiation 68 (CD68) and toll-like receptor 4 levels (TLR4) in liver issue in normal control (NC), model control (MC), positive control (Essentiale) and *Linderae radix* ethanol extract (LREE) groups.

The expression levels of TLR4 and CD68 increased in the model group *vs* the normal group (Figure 4). Compared with their levels in the model group, the expression levels of TLR4 and CD68 proteins were downregulated in the LGM and LGH groups.

### LREE promoted the expressions of tight junction proteins in the small intestine

**Figure 5.**
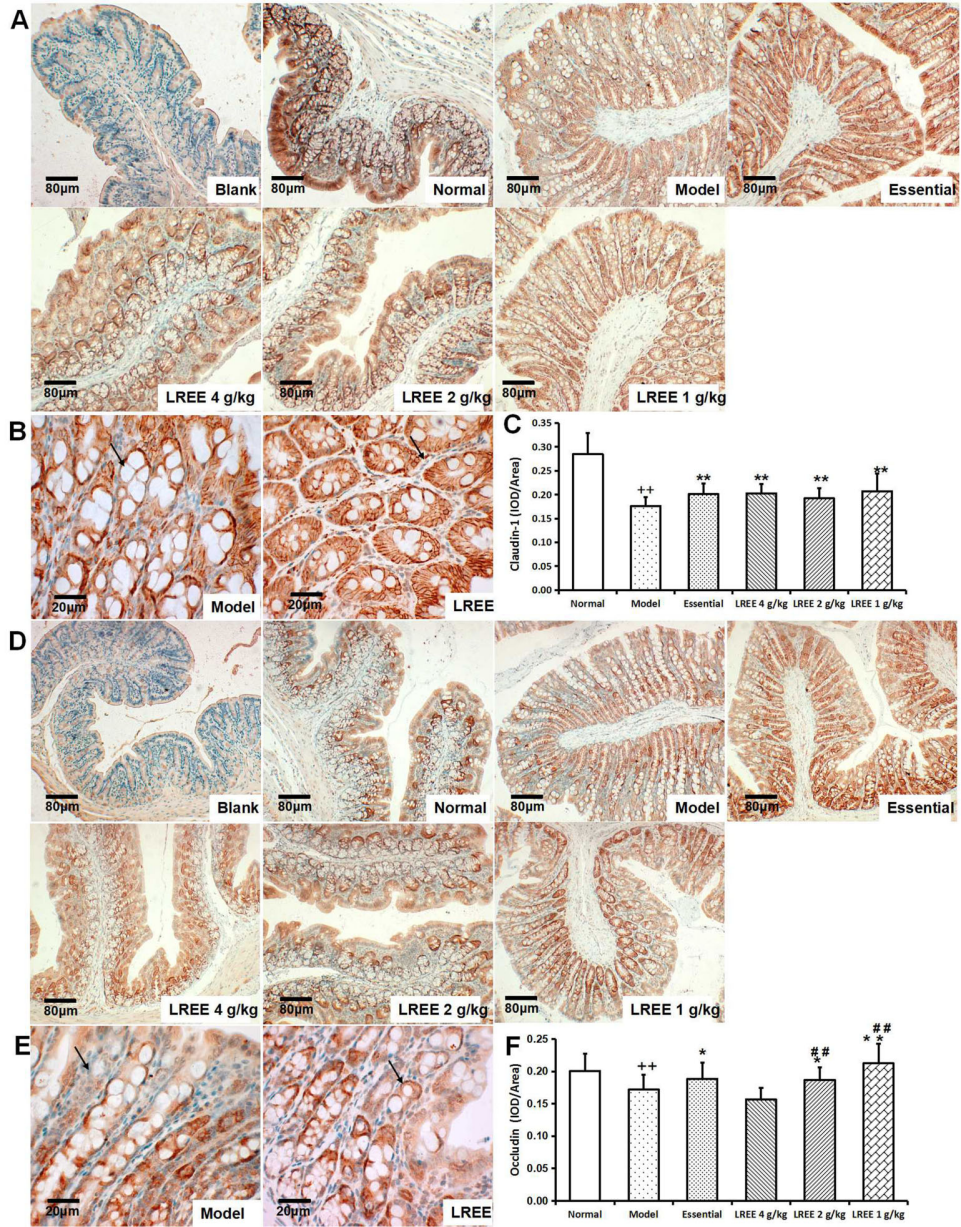
Immunohistochemical analysis of claudin-1 and occludin protein levels in the small intestine (3,3′-diaminobenzidine staining). Claudin-1 staining in the different groups was observed under a fluorescence microscope at 100× (**A**, bar: 80 μm) and 400× magnification (**B**, bar: 20 μm). **C**, relative quantitative expression of claudin-1. Occludin staining in the different groups was observed under a microscope at 100× (**D**, bar: 80 μm) and 400× magnification (**E**, bar: 20 μm). **F**: relative quantitative expression of occludin. To induce alcoholic liver disease, 50% alcohol was provided, and *Linderae radix* ethanol extract (LREE) was administered daily by gavage for the same time. The expression of occludin and claudin-1 was significantly enhanced (dark brown color). Data are reported as means±SD.^++^P*<*0.01 *vs* the normal control group; *P<0.05 and **P<0.01 *vs* the model control group; *^##^*P<0.01 *vs* the high concentration of LREE group (ANOVA).

The expression levels of claudin-1 (Figure 5A and B) and occludin (Figure 5D and E) in different groups were detected. As shown in Figure 5B and D, claudin-1 and occludin were disrupted irregularly in the intestine tissue of rats exposed to alcohol. Protein levels of both claudin-1 and occludin were significantly decreased (Figure 5C and F) and the localization of occludin was modified. The expression levels of claudin-1 and occludin were increased by LREE administration.

## Discussion

In the present study, an ALD model rat was established. Serum levels of ALT, IL-6, NF-κB, and portal vein LPS levels in the model rats were significantly increased compared with those in the normal group. Meanwhile, inflammatory cell infiltration and associated necrotic changes in liver tissue were found on histopathology observation. In addition, the abundance of Firmicutes was reduced and the abundance of Bacteroidetes was increased in the model group. By contrast, LREE treatment increased the number of Firmicutes but decreased the number of Bacteroidetes. Furthermore, the overexpression of TLR4 in liver tissue was effectively suppressed and the levels of tight junction proteins in intestine tissue were enhanced by LREE administration.

Disordered lipid metabolism and transaminase activity changes are present in alcoholic liver injury (28), and ALT, AST, and T.BIL are the major makers for the diagnosis of ALD. In the present study, we measured the ALT, AST, and T.BIL levels to evaluate the liver injury of the model rats. The levels of ALT and T.BIL were significantly increased in model rats compared with those in normal rats; however, there was no change in AST. An increased ALT level is a sensitive indicator of liver injury and ALT is mainly released in liver cell cytoplasm while the increased AST is mainly released in hepatocellular mitochondria and could reflect the severity of liver injury (29). In the early ethanol-induced liver-injured rats, the liver cell membrane is destroyed first, and hepatocellular mitochondria is damaged in late stage of liver injured rats (29). Combined with the HE staining results, our results suggested that ALD model rats had a mild ethanol-induced liver injury.

Alcohol and its metabolites break the balance of intestinal microorganisms, including the increased number of gram-negative bacteria, which produce endotoxins (30). Generally, gut microbiota regulate the metabolic barrier function of the gut (31). In patients with alcoholic psychosis, higher activities of ALT and AST were discovered and a lower abundance of enterococci, lactobacilli, and bifidobacteria was observed (31). Probiotic therapy could decrease the level of liver enzymes (31). Cai et al. (32) revealed a lower abundance of Bacteroidetes and a higher abundance of Firmicutes in nucleotides-treated rats than in those treated with alcohol. In another study, Bull-Otterson et al. (33) showed that the abundance of both Bacteroidetes and Firmicutes decreased during chronic ethanol feeding. Another study revealed that the abundance of Firmicutes was decreased and the abundance of Bacteroidetes was increased in alcohol-treated mice (10). In this study, alcohol treatment resulted in a significant reduction in the Shannon diversity index compared with that in the Essentiale and LREE groups. Taken together, chronic alcohol treatment induced distinct changes in the gut microbiota, whereas LREE had a prebiotic effect on the balance of the gut microbiota induced by alcohol.

Gut-derived LPS recruits macrophages via TLR4 to overexpress inflammatory cytokines such as IL-6, TNF-α, which contribute to the inflammatory processes in alcoholic liver disease (34). In the present study, overexpression of TLR4 and inflammatory cells with necrosis were found in the livers of rats treated with alcohol. Meanwhile, IL-6, TNF-α, and NF-κB levels in systemic circulation increased significantly. These findings agreed with those of previous studies that alcohol contributes to increase TLR4, which mediates alcohol-induced liver damage, including alcoholic hepatitis (35). The results of the current study showed that LREE could block the increase in serum cytokine levels, downregulate the expression of TLR4 in the liver, and ameliorate liver tissue pathological changes, indicating LREE has a good anti-inflammatory effect related to regulation of the TLR4-mediated signaling pathway, which agreed with the results of the study by Cai et al. (32).

In addition, we explored the anti-inflammatory effect of LREE at different concentrations. As expected, the serum levels of IL-6 and IL-8 were reduced in a dose-dependent manner in the LGL, LGM, and LGH groups; however, the TNF-α and NF-κB levels did not show a linear dose-response relationship in the different LREE concentrations groups and were only significantly decreased in the LGM group. One possible explanation was that LREE is a Chinese herb extract with many components and is not a pure compound (36); it is possible that some unknown components of LREE may influence the anti-inflammatory effects at higher concentrations, but it is still unclear and needs to be explored by future experiments. In addition, high concentration inhibition is observed in similar studies of Chinese herbs treatment (37,38), which reveals the therapeutic effect is significantly improved under low concentration treatment, but is significantly reversed or reduced after high concentration treatment. The drugs at different doses may selectively activate or inhibit different signaling molecules (38). Therefore, each drug may have its optimum concentration point and selection of concentration range may directly influence the therapeutic effect.

Tight junction (TJ) proteins between intestinal epithelial cells, such as occludin, claudin-1, and zona-occludens-1 are components that maintain the integrity of the intestinal mucosa (39). Alcohol has both direct and indirect effects on the TJ functions and its metabolites (40). In this study, after intragastric administration of LPS for 3.5 h, higher hepatic portal vein LPS levels were found in rats with alcohol intake compared with normal control rats. Transmission electron microscopy also showed that the intestinal ultrastructure was impaired, and the decreased expression level of claudin-1 and occludin in the intestinal tissue confirmed TJ dysfunction, which would lead to decreased intestinal permeability. LREE increased the expression levels of TJ proteins, improved intestinal ultrastructure, and decreased portal vein LPS levels, suggesting that LREE could block intestinal endotoxemia via intestinal barrier protection.

In conclusion, LREE had a protective effect on the intestinal barrier, attenuating the disturbance of gut microbiota and alleviating the intestinal endotoxemia associated with alcoholic liver injury. However, these results should be verified via further experiments.

## References

[1] Orman ES, Odena G, Bataller R (2013). Alcoholic liver disease: pathogenesis, management, and novel targets for therapy. J Gastroenterol Hepatol.

[2] Organization WH (2014). Global status report on alcohol and health 2014. Global Status Report on Alcohol.

[3] Abnet CC, Chen Y, Chow WH, Gao YT, Helzlsouer KJ, Le Marchand L (2010). Circulating 25-hydroxyvitamin D and risk of esophageal and gastric cancer: cohort consortium vitamin D pooling project of rarer cancers. Am J Epidemiol.

[4] Schwartz JM, Reinus JF (2012). Prevalence and natural history of alcoholic liver disease. Clin Liver Dis.

[5] Coombes JD, Syn WK (2016). Pathogenic mechanisms in alcoholic liver disease (ALD): Emerging Role of Osteopontin. Molecular Aspects of Alcohol and Nutrition.

[6] Fleming S, Toratani S, Shea-Donohue T, Kashiwabara Y, Vogel SN, Metcalf ES (2001). Pro- and anti-inflammatory gene expression in the murine small intestine and liver after chronic exposure to alcohol. Alcohol Clin Exp Res.

[7] Szabo G, Mandrekar P, Petrasek J, Catalano D (2011). The unfolding web of innate immune dysregulation in alcoholic liver injury. Alcohol Clin Exp Res.

[8] Rocco A, Compare D, Angrisani D, Zamparelli MS, Nardone G (2014). Alcoholic disease: liver and beyond. World J Gastroenterol.

[9] Cani PD, Delzenne NM (2007). Gut microflora as a target for energy and metabolic homeostasis. Curr Opin Clin Nutr Metab Care.

[10] Yan AW, Fouts DE, Brandl J, Stärkel P, Torralba M, Schott E (2011). Enteric dysbiosis associated with a mouse model of alcoholic liver disease. Hepatology.

[11] Steven RG, Mihai P, Robert TD, Paul BE, Peter JT, Buck SS (2006). Metagenomic analysis of the human distal gut microbiome. Science.

[12] Casafont Morencos F, de las Heras Castaão G, Martín Ramos L, López Arias MJ, Ledesma F, Pons Romero F (1996). Small bowel bacterial overgrowth in patients with alcoholic cirrhosis. Dig Dis Sci.

[13] Keshavarzian A, Farhadi A, Forsyth CB, Rangan J, Jakate S, Shaikh M (2009). Evidence that chronic alcohol exposure promotes intestinal oxidative stress, intestinal hyperpermeability and endotoxemia prior to development of alcoholic steatohepatitis in rats. J Hepatol.

[14] Wang HJ, Gao B, Zakhari S, Nagy LE (2012). Inflammation in alcoholic liver disease. Annu Rev Nutr.

[15] Mandrekar P, Szabo G (2009). Signalling pathways in alcohol-induced liver inflammation. J Hepatol.

[16] Neuman MG, Maor Y, Nanau RM, Melzer E, Mell H, Opris M (2015). Alcoholic liver disease: role of cytokines. Biomolecules.

[17] Wu Y, Zheng Y, Liu X, Han Z, Ren Y, Gan L (2010). Separation and quantitative determination of sesquiterpene lactones in Lindera aggregata (wu-yao) by ultra-performance LC-MS/MS. J Sep Sci.

[18] Luo Y, Liu M, Yao X, Xia Y, Dai Y, Chou G (2009). Total alkaloids from Radix Linderae prevent the production of inflammatory mediators in lipopolysaccharide-stimulated RAW 264.7 cells by suppressing NF-kappaB and MAPKs activation. Cytokine.

[19] Wang C, Dai Y, Yang J, Chou G, Wang Z (2007). Treatment with total alkaloids from Radix Linderae reduces inflammation and joint destruction in type II collagen-induced model for rheumatoid arthritis. J Ethnopharmacol.

[20] Yamahara J, Matsuda H, Sawada T, Kushida H (1983). Biologically active principles of crude drugs preventive effect of sesquiterpenoid components of the root of *Lindera strychinifolia* on experimental liver damage [in Japanese]. Japan J Pharmacognosy.

[21] Zhu M, Luk CT, Lew TH (2008). Cytoprotective effect of lindera aggregata roots against ethanol-induced acute gastric injury. Pharma Biol.

[22] Wang JW, Chen XY, Hu PY, Tan MM, Tang XG, Huang MC (2016). Effects of Linderae radix extracts on a rat model of alcoholic liver injury. Exp Ther Med.

[23] Wang Y, Liu L, Zhang G, He Z, Lou Z (2018). A study on the dosage and function association of ethanol radix linderate extraction against chronic alcoholic liver injury [in chinese]. Zhejiang Clin Med J.

[24] Caporaso JG, Kuczynski J, Stombaugh J, Bittinger K, Bushman FD, Costello EK (2010). QIIME allows analysis of high-throughput community sequencing data:. Nat Methods.

[25] Maidak BL, Olsen GJ, Larsen N, Overbeek R, Mccaughey MJ, Woese CR (2000). The RDP (Ribosomal Database Project). Nucleic Acids Res.

[26] Edgar RC (2010). Search and clustering orders of magnitude faster than BLAST. Bioinformatics.

[27] Mullins TD, Giovannoni SJ (1995). Genetic comparisons reveal the same unknown bacterial lineages in Atlantic and Pacific bacterioplankton communities. Limnology and Oceanography.

[28] Roychowdhury S, Mcmullen MR, Pisano SG, Liu X, Nagy LE (2013). Absence of receptor interacting protein kinase 3 prevents ethanol-induced liver injury. Hepatology.

[29] Sai GU (2006). Establishment of experimental model of chronic alcoholic fatty liver in rats [in chinese]. Journal of Chongqing Medical University.

[30] Purohit V, Bode JC, Bode C, Brenner D, Choudhry M, Hamilton F (2008). Alcohol, intestinal bacterial growth, intestinal permeability to endotoxin, and medical consequences: summary of a symposium. Alcohol.

[31] Compare D, Coccoli P, Rocco A, Nardone OM, De MS, Carten M (2012). Gut--liver axis: the impact of gut microbiota on non-alcoholic fatty liver disease. Nutr Metab Cardiovasc Dis.

[32] Cai X, Bao L, Wang N, Ren J, Chen Q, Xu M (2016). Dietary nucleotides protect against alcoholic liver injury by attenuating inflammation and regulating gut microbiota in rats. Food Funct.

[33] Bull-Otterson L, Feng W, Kirpich I, Wang Y, Qin X, Liu Y (2013). Metagenomic analyses of alcohol induced pathogenic alterations in the intestinal microbiome and the effect of Lactobacillus rhamnosus GG treatment. PLoS One.

[34] Petrasek J, Mandrekar P, Szabo G (2010). Toll-like receptors in the pathogenesis of alcoholic liver disease. Gastroenterol Res Pract.

[35] Inokuchi S, Tsukamoto H, Park E, Liu ZX, Brenner DA, Seki E (2011). Toll-like receptor 4 mediates alcohol-induced steatohepatitis through bone marrow-derived and endogenous liver cells in mice. Alcohol Clin Exp Res.

[36] Wu YJ, Zheng YL, Luan LJ, Liu XS, Han Z, Ren YP (2015). Development of the fingerprint for the quality of Radix Linderae through ultra-pressure liquid chromatography-photodiode array detection/electrospray ionization mass spectrometry. J Sep Sci.

[37] Wang YC, Lin XY, Wang F, Liao HL, Liu W, Chen MY (2015). Effects of imperatorin and isoimpemtorin on biological activity and migration of human epidermal melanocytes [in Chinese]. Chinese Journal of Dermatovenereology.

[38] Dolinsky VW, Soltys CL, Rogan KJ, Chan AY, Nagendran J, Wang S (2015). Resveratrol prevents pathological but not physiological cardiac hypertrophy. J Mol Med.

[39] Schnabl B, Brenner DA (2014). Interactions between the intestinal microbiome and liver diseases. Gastroenterology.

[40] Elamin E, Juuti-Uusitalo K, van IJzendoorn S, Troost F, Duimel H, Broers J (2012). Effects of ethanol and acetaldehyde on tight junction integrity: in vitro study in a three dimensional intestinal epithelial cell culture model. PLoS One.

